# COVID-19 confines recreational gatherings in Seoul to familiar, less crowded, and neighboring urban areas

**DOI:** 10.1057/s41599-022-01349-4

**Published:** 2022-09-23

**Authors:** Jisung Yoon, Woo-Sung Jung, Hyunuk Kim

**Affiliations:** 1 Kellogg School of Management at Northwestern University, Evanston, IL 60208 USA; 2 Northwestern Institute on Complex Systems, Evanston, IL 60208 USA; 3 Department of Industrial and Management Engineering, Pohang University of Science and Technology, Pohang, 37673 Republic of Korea; 4 Department of Physics, Pohang University of Science and Technology, Pohang, 37673 Republic of Korea; 5 Department of Administrative Sciences, Metropolitan College, Boston University, Boston, MA 02215 USA

**Keywords:** Information systems and information technology, Complex networks, Geography

## Abstract

Recreational gatherings are sources of the spread of infectious diseases. Understanding the dynamics of recreational gatherings is essential to building effective public health policies but challenging as the interaction between people and recreational places is complex. Recreational activities are concentrated in a set of urban areas and establish a recreational hierarchy. In this hierarchy, higher-level regions attract more people than lower-level regions for recreational purposes. Here, using customers’ motel booking records which are highly associated with recreational activities in Korea, we identify that recreational hierarchy, geographical distance, and attachment to a location are crucial factors of recreational gatherings in Seoul, Republic of Korea. Our analyses show that after the COVID-19 outbreak, people are more likely to visit familiar recreational places, avoid the highest level of the recreational hierarchy, and travel close distances. Interestingly, the recreational visitations were reduced not only in the highest but also in low-level regions. Urban areas at low levels of the recreational hierarchy were more severely affected by COVID-19 than urban areas at high and middle levels of the recreational hierarchy.

## Introduction

Human urban activities are principal elements of social phenomena, including the growth of cities (Bettencourt and Zünd, 2020; Verbavatz and Barthélemy, 2020), economies (Park et al., 2019; Storz et al., 2015), and epidemics (Chang et al., 2021). They tend to be concentrated in parts of cities and form a hierarchy of geographical areas, where regions at upper levels attract more people than those at lower levels (Barthélemy, 2016; Batty, 2013; Pan et al., 2013). A person may frequently visit a popular region, often referred to as a *hotspot* (Bassolas et al., 2019; Louail et al., 2015; Roth et al., 2011), even though it is far from living areas.

Strong urban hierarchy raises various concerns during a pandemic (Ahmed et al., 2020; van Dorn et al., 2020; Yu et al., 2021). The spread of infectious diseases would be broad and prevalent if it originates from a hotspot at the top of the hierarchy (Albert et al., 2000; Gould and Wallace, 1994; Kang et al., 2020; Pastor-Satorras and Vespignani, 2003). The economic impact of a pandemic also differs by hierarchy level. The income of the populations working in the informal economy, which is usually located at low hierarchy levels, was negatively affected by the COVID-19 pandemic (Narula, 2020). Despite its importance to human activities, the urban hierarchy has been rarely considered when analyzing behavioral changes in response to a pandemic (Batty et al., 2021; Moro et al., 2021; Nouvellet et al., 2021; Schläpfer et al., 2021; Song et al., 2010).

Here, by using individual-level motel booking records (see the “Methods” section) from a leading Korean accommodation platform, we compare a visitation pattern before and after the COVID-19 outbreak. According to a market report in 2021 by Dighty (2021), 32.6% of the platform’s mobile application installers were in their 20s, 35.4% were in their 30s, 23.9% were in their 40s, and 6.4% were above 50s. Additionally, 37.7% were females, and 63.3% were males. Therefore, low- and middle-income populations are likely to be the primary users of the platform and the Korean accommodation market.

Motels are often located near recreational places such as pubs, nightclubs, restaurants, and cafes in urban hotspots (Lashley, 2016). Especially, in recent years that our data cover, the Korean motel industry has transformed itself into an entertainment industry that provides physical spaces for relaxation and cultural activities (Kim and Kim, 2018). Young Koreans increasingly book motels for intimate relationships and partying with friends because motels are more affordable and accessible than hotels (Hu, 2016). For these reasons, we use our motel reservation data as a proxy for recreational gatherings in Seoul. The hierarchy of recreational urban areas which is extracted from our motel booking data is referred to as the *recreational hierarchy*. Our analyses show that recreational hierarchy, geographical distance, and attachment to a location are important factors of recreational gatherings in Seoul, the largest city in the Republic of Korea.

## Methods

### Accommodation reservation data

We sourced an accommodation reservation data set from Goodchoice Company LTD, a Korean platform that occupied 29% of the online market share in 2020 (Wiseapp Report, https://www.dailypop.kr/news/articleView.html?idxno=51946, a news article written in Korean). The data contain anonymized customer-level reservation histories, spanning the period from January 2019 to November 2020, and geographic locations of 1038 unique motels in Seoul, Republic of Korea. No demographic information is available.

### Seoul mobility data

Seoul mobility data was downloaded from the Seoul Open Data Plaza (https://data.seoul.go.kr/dataVisual/seoul/seoulLivingMigration.do, Accessed on 3 December 2021). The data contains mobility flows between administrative divisions (425 divisions in total) decomposed by gender, age, time of departure, and time of arrival, by aggregating the mobile phone signals from transceiver stations in Seoul. Also, for each individual, the data provide an estimated daytime residence (denoted as “W”, mostly workplace), nighttime residence (denoted as “H”, mostly home), and other classes (denoted as “E”). With the classifications above, we can infer the context of urban mobility. For instance, a movement from a workplace to another area to enjoy recreational gatherings is classified as the “WE” type. We use the mobility data spanning the period from January 2020 to October 2020 and focus on the mobility types with “WE”, “HE”, and “EE” to track non-routine mobility patterns for recreational gatherings.

### Hotspot entropy and the radius of recreational activities

Based on the recreational hierarchy, we characterize location trajectories with two proposed measures: *hotspot entropy* and *radius of recreational activities*. First, the hotspot entropy is the Shannon entropy of hotspot levels in a trajectory. It is defined as

1 $$h=-\mathop{\sum }\limits_{i=1}^{L}{p}_{i}\log {p}_{i},$$

where *L* is the total number of hotspot levels (*L* = 10) and *p*_*i*_ is the frequency of a hotspot level *i* in the trajectory. For example in Fig. 4a, *p* is $[\frac{2}{3},\frac{1}{6},\frac{1}{6},0,0,0,0,0,0,0]$ and hotspot entropy *h* is $-\frac{2}{3}\log \frac{2}{3}-\frac{1}{6}\log \frac{1}{6}-\frac{1}{6}\log \frac{1}{6}=0.867$.

Second, to quantify how far the places are in a trajectory, we define the radius of recreational activities. It is the variance of geographic distances from the most frequent cell (home cell) in a trajectory (similar to the radius of gyration) and is calculated as

2 $$r=\sqrt{\frac{\mathop{\sum }\nolimits_{i = 1}^{N}{d}_{i,{\rm {home}}}^{2}}{N}},$$

where *N* is the length of a trajectory, *d* is the Haversine distance between the centers of the two cells, and home is the *recreational home* cell that is the most frequent cell in the trajectory. If there are multiple most frequent cells, we randomly pick one as the home cell.

## Results

**Fig. 1:**
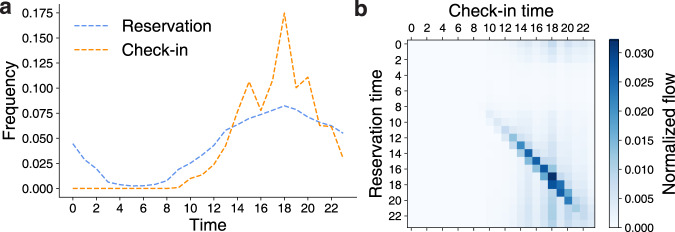
Motel reservation and check-in times distributions. **a** Reservation and check-in time distributions. Reservations and check-in take place primarily after 12 p.m. **b** A heatmap for the reservation and check-in times. Both reservation and check-in times show a similar pattern and are concentrated after 12 p.m.

**Fig. 2:**
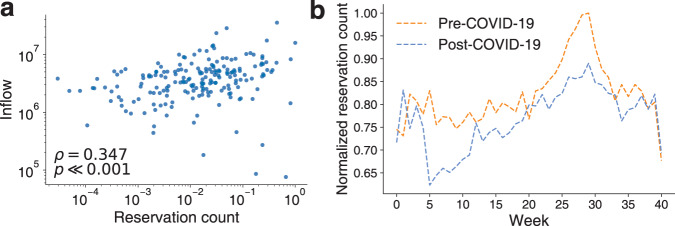
Motel reservations as a proxy for recreational gatherings in Seoul. **a** A comparison of the reservation counts to the mobility inflows in Seoul, Republic of Korea. We aggregate the mobility inflows at the level of the administrative division. Each dot represents an administrative division. A significant correlation supports that the reservation history data can be a good proxy for urban recreational gatherings in Seoul. **b** Weekly reservation counts. For a data privacy concern, we normalize the weekly reservation counts by the maximum weekly reservation count.

In our data, motel reservation and check-in times are concentrated after 12 p.m. (Fig. 1a) and there is little time difference between reservation and check-in times (Fig. 1b). To validate whether our data capture recreational gatherings in Seoul to some extent, we compare reservation counts at the administrative division level with the mobility inflows from Seoul mobility data aggregating GPS locations (see the “Methods” section). The rank correlation between the reservation counts and the mobility inflows is significant (*ρ* = 0.347, *p*-value < 0.001; Fig. 2a). The correlation becomes stronger if we only consider nighttime inflows (from 9 p.m. to 6 a.m., *ρ* = 0.400, *p*-value < 0.001).

To understand the effect of COVID-19 on recreational gathering behaviors, we split the data into two periods: pre-COVID-19 (From January 21, 2019 to November 3, 2019) and post-COVID-19 (From January 20, 2020 to November 1, 2020). January 20, 2020 is the first day that a COVID-19 infection case was reported in Korea. Both periods start from the fourth week of January and span 286 days. The weekly trend of reservation counts is shown in Fig. 2b. A significant drop appears near Week 5, the first week of the official social distancing in the Republic of Korea. The reservation counts recovered gradually to the normal state even after several restrictions were imposed.

### Recreational hierarchy of Seoul

**Fig. 3:**
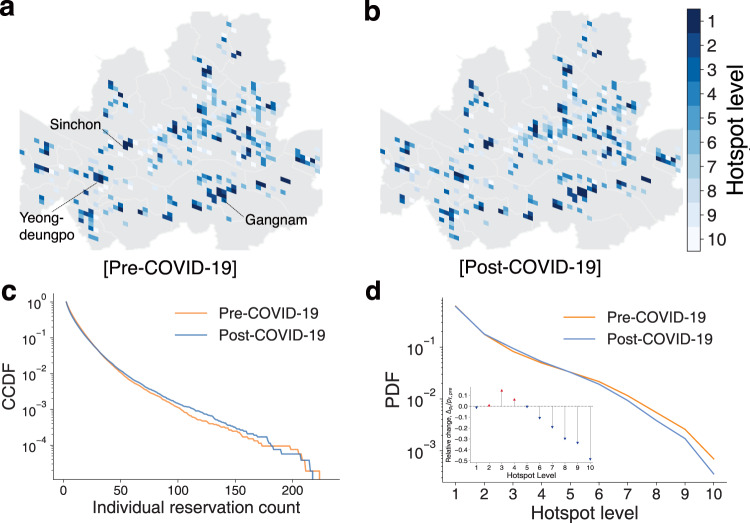
Recreational hierarchy of Seoul. **a** and **b** The pre- and post-COVID-19 recreational hierarchy maps. Each cell represents a level-14 Google S2 cell colored by its hotspot level. The areas with gray color represent cells with no accommodations. **c** The complementary cumulative distribution function (CCDF) of individual reservation counts. On average, individual reservation counts decrease after the COVID-19 outbreak. **d** The reservation count distribution by the hotspot level *p*_*ℓ*_. The inset shows the relative change of *p*_*ℓ*_, (*p*_*ℓ*,post_−*p*_*ℓ*,pre_)/*p*_*ℓ*,pre_. A red up-arrow indicates an increase in the probability and a blue down-arrow indicates a decrease in the probability compared to the distribution for the pre-COVID-19 period.

We assign each motel to a Google S2 cell (https://github.com/google/s2geometry). S2 cells are space tessellations that divide the Earth into cells of a similar size area. It is known as a robust, flexible spherical geometry (Bassolas et al., 2019; Veach et al., 2017; Wu et al., 2018). We used level-14 S2 cells whose size ranges from 0.19 to 0.40 km^2^ (on average 0.32 km^2^). Then, we aggregate the reservation counts by S2 cell and identify a hierarchy of cells by assigning a *hotspot level*, an inverse decile rank of aggregated reservation counts, to each cell. Level 1 is the highest, and level 10 is the lowest level. Figure 3a, b show the recreational hierarchy maps for both periods. The assigned hotspot levels are almost consistent for both periods. Cells with high levels correspond to popular recreational areas in Seoul such as Gangnam, Sinchon, and Yeongdeungpo Time Square (highlighted in Fig. 3a).

To further analyze behavioral changes induced by COVID-19, we take a subset of customers who have at least two reservation records in both the pre- and post-COVID-19 periods as the focus group. This focus group covers 30% of the total customers in the pre-COVID-19 period and 26% in the post-COVID-19 period. The distributions of individual reservation counts are similar for both periods, but the average individual reservation counts decreased after the COVID-19 outbreak (Fig. 3c; 〈*l*_pre_〉 = 9.200 > 〈*l*_post_〉 = 8.757; paired *t*-statistic = 8.820, *p*-value ≪ 0.001).

As shown in Fig. 3d, the majority of reservations (61.6% for the pre-COVID-19 period, 60.7% for the post-COVID-19 period) are concentrated in the top 10% of cells, while the bottom 10% of cells only have a few reservations (0.6% for the pre-COVID-19 period, 0.3% for the post-COVID-19 period), suggesting the inequality of recreational visitations on urban areas for both periods. Interestingly, COVID-19 affects the inequality of urban areas differently by the hierarchy level. The proportion of the highest level decreases after the COVID-19 outbreak (Fig. 3d inset). However, this proportion was not equally distributed across other levels. People visited levels 2, 3, and 4 rather than low levels (*l* ≤ 6). Our findings show that the COVID-19 pandemic worsened the inequality across urban areas, in line with previous studies on income levels (Belot et al., 2021; Zhang et al., 2022) and costs of shutdown (Hevia et al., 2022). We explain the worsening inequality by decomposing individual recreational gathering behaviors in the next section.

### Factors of recreational gatherings

**Fig. 4:**
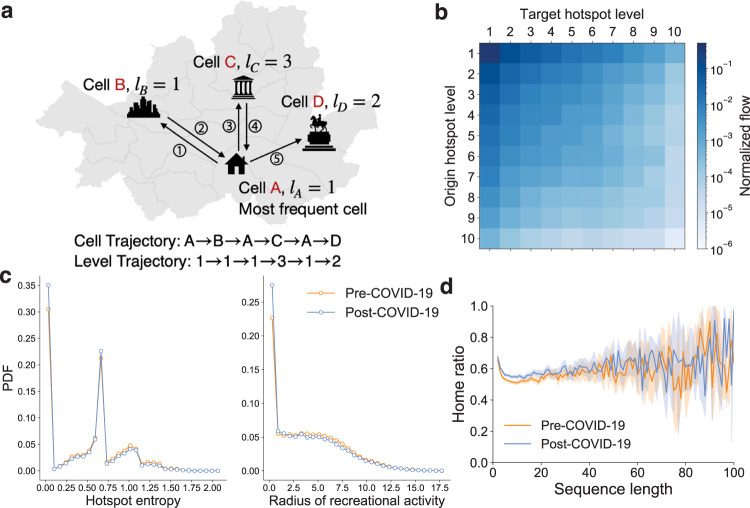
The factors of recreational gatherings: hierarchy, geographical distance, and attachment to a location. **a** An illustrative example of the cell and level trajectories. **b** The flow matrix *T*^data^ for the pre-COVID-19 period. The trips within the same cell are excluded. We also provide the flow matrix for the post-COVID-19 period in Supplementary Information (Fig. S1). **c** The distributions of the hotspot entropy ${p}_{{\mathrm {h}}}^{{\mathrm {data}}}$ and the radius of recreational activities ${p}_{{\mathrm {r}}}^{{\mathrm {data}}}$ for both periods. After the outbreak, people explored less across the recreational hierarchy. **d** Home ratios by sequence length for both periods. Attachment to a location appears regardless of sequence length, implying the share of time in the recreational home remains constant after the outbreak.

Individual records can be converted to sequences of cells and hotspot levels. The arrows in Fig. 4a represent a synthetic journey that consists of urban areas. The *cell trajectory* of this example is *A* → *B* → *A* → *C* → *A* → *D*. Note that the same place can appear multiple times. Based on the assigned levels of the cells, the *level trajectory* is 1 → 1 → 1 → 3 → 1 → 2. For each trajectory constructed from the data, we define the most frequent cell as the *recreational home*, so *A* is the home in the example.

#### Hierarchy: Transition between levels

We construct a flow matrix *T*^data^ where ${T}_{ij}^{{\rm {data}}}$ is the number of trips between level *i* and *j* normalized by the total flows (Fig. 4b). We here exclude self-transitions, trips within the same cell, to focus on the transitions between different cells. The majority of transitions are concentrated at high levels of the hierarchy, and the flow matrix is almost symmetric. To check whether the transition from level *i* to level *j*, *p*(*j*∣*i*), depends on level *j*, we build a null model Bassolas et al. (2019) that *p*(*j*∣*i*) is proportional to the total inflows to the destination’s level *j* as follows:

3 $${T}_{ij}^{{\rm {null}}}=\mathop{\sum }\limits_{k=1}^{L}{T}_{ik}\frac{\mathop{\sum }\nolimits_{m = 1}^{L}{T}_{mj}}{\mathop{\sum }\nolimits_{m,k = 1}^{L}{T}_{mk}},$$

where $\mathop{\sum }\nolimits_{k = 1}^{L}{T}_{ik}$ is the total outflow from level *i* and $\mathop{\sum }\nolimits_{m = 1}^{L}{T}_{mj}/\mathop{\sum }\nolimits_{m,k = 1}^{L}{T}_{mk}$ is the fraction of the inflows to level *j* (Supplementary Fig. S2). Comparing the ratio of *T*^data^ to *T*^null^ (Supplementary Fig. S3), we confirm that *T*^data^ is close to *T*^null^ at high levels of the hierarchy, while the ratios of the transitions from or to low levels of the hierarchy increase. Most of the transitions are at high levels of the hierarchy, and inflow and outflow are symmetric (Supplementary Fig. S4; *R*^2^ = 0.99 for both periods). Hence, transitions between hotspot levels are approximately independent of the previous place’s hotspot level *p*(*j*∣*i*) ≃ *p*(*j*) which follows the reservation count distribution by the hotspot level *p*_*ℓ*_.

#### Hierarchy: hotspot entropy

To measure the extent to which hotspot levels are diverse in individual records, we introduce the hotspot entropy, *h* (see the “Methods” section). *h* is zero if the trajectory consists of cells of the same hotspot level. The maximum value of *h* is $\ln {{\mbox{(number of hotspot levels)}}}\,=\ln 10$. The distributions of *h* for both periods are different (KS-statistic = 0.046, *p*-value ≪ 0.001) and shown in Fig. 4c (left). Before the COVID-19 outbreak, 30% of people stay only at a single level on average, while this proportion increases after the outbreak. Also, the mean hotspot entropy decreases (〈*h*_pre_〉 = 0.518 > 〈*h*_post_〉 = 0.475), implying people are less likely to visit different levels.

#### Geographical distance

The *radius of recreational activities*, *r* (see the “Methods” section), a variance of geographical distances from the recreational home of a sequence, quantifies how far the places in a trajectory are. The unit of *r* is a kilometer (km). The distributions of *r* show that the majority of people stay within a single cell without moving to other cells (Fig. 4c, right). The likelihood of visiting distant places is inversely proportional to geographical distance, while the hierarchy leads people to move farther than expected (Supplementary Fig. S5). Considering the radius of the biggest district in Seoul (Seocho district) is about 5.523 km, we can say that more than 33% of the platform users in the pre-COVID-19 period visit places outside the home cell (~30% for the post-COVID-19 period). Overall, *r* decreases after the COVID-19 outbreak (〈*r*_pre_〉 = 4.094 > 〈*r*_post_〉 = 3.738), and the distributions for both periods are significantly different (KS-statistic = 0.056, *p*-value ≪ 0.001). This evidence suggests that people tend to stay close to their recreational homes after the outbreak.

#### Attachment to a location

Attachment to a location is an indicator of customer satisfaction and an important factor for the accommodation business (Bowen and Chen, 2001; Kandampully and Suhartanto, 2000). In Fig. 4d, we show the home ratio which is the fraction of the most frequent cell in a sequence. Interestingly, the home ratio is about 0.6 regardless of sequence length. The home ratio slightly increases after the COVID-19 outbreak for the light users who booked motels no more than 20 times, while there is no difference in the home ratio between the two periods for the heavy users who booked motels more than 20 times (top 10% users by sequence length).

### A model for replicating reservation records

**Fig. 5:**
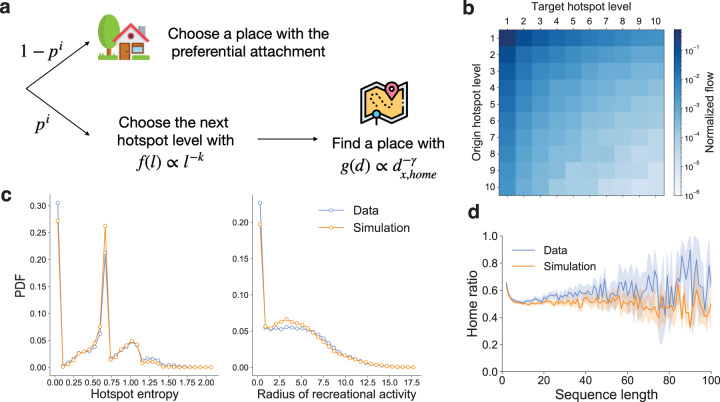
A mechanistic model for detecting behavioral changes. **a** A schematic diagram of our model. *k*, *γ*, and *p* control the effects of recreational hierarchy, geographical distance, and attachment to a location, respectively. *i* is the index of an iteration. **b** The flow matrix of the best model. **c** The distributions of the hotspot entropy distribution *p*_h_ (left) and the radius of recreational activities *p*_r_ (right). Blue lines are the empirical distributions, and orange lines are the simulation results. **d** Home ratios by sequence length. Here, we show the results from the best model for the pre-COVID-19 period. The best model for the post-COVID-19 period is in Supplementary Fig. S6.

Our empirical analysis reveals that recreational hierarchy, geographical distance, and attachment to a location need to be considered simultaneously to explain recreational gatherings in Seoul, Republic of Korea. Leveraging our key findings on the individual movements, we develop a model reproducing their patterns and detecting behavioral changes during the COVID-19 pandemic (Fig. 5a). Our model is motivated by the literature analyzing human mobility (Moro et al., 2021; Schläpfer et al., 2021; Song et al., 2010). First, an agent starts from an initial cell randomly picked from the cell-level reservation count distribution. Then, the agent explores places with probability *p*_*i*_ or chooses a previously visited place in proportion to the frequency in the reservation history with probability 1−*p*_*i*_, where *p* ∈ [0, 1] controls the likelihood that the agent decides to explore places and *i* is the number of iterations starting from one. As iteration *i* increases, the agent is more likely to choose previously visited places.

If the agent decides to explore places, the agent first chooses the hotspot level for the next move from the distribution $f\left(\ell \right)\propto {\ell }^{-k}\,(k\in \left[0\right.,\infty )$). *k* can be interpreted as the preference for high hierarchy levels of the agent. For instance, if *k* = 0, the agent randomly selects the next level without considering recreational hierarchy. On the other hand, with a large *k*, most reservations are concentrated in high hierarchy levels. Next, the agent chooses a place of the given level in proportion to the inverse of geographical distance with an exponent $\gamma \in \left[0,\infty \right)$ which controls the likelihood of visiting distant places from the recreational home. If *γ* = 0, the agent ignores the geographical distance and randomly picks the place with the given level. In this step, the agent can pick the recreational home by imputing the *d*_home,home_ = 1, and the recreational home cell can change according to the current history of the agent as the iteration proceeds. The steps above are repeated until we have a sequence of which length is equal to the length of the original trajectory. We keep the sequence length distribution from the data (Fig. 3c) to control the effect of sequence length.

Through a grid search, we estimate the model parameters that minimize the Jensen–Shannon divergence (JSD) of the hotspot entropy distribution *p*_h_, the reservation count distribution *p*_l_, and the radius of recreational activities distribution *p*_r_ between synthetic sequences and the data. Note that *p* and *k* affect *p*_l_ and *p*_h_, while *γ* is independent of *p*_l_ and *p*_h_. Taking advantage of this property, we jointly optimize the model by searching for the best *p* and *k* that minimize the product of JSD of *p*_l_ and *p*_h_, namely JSD_Entropy_ and JSD_Hierarchy_. Next, with the best *p* and *k*, we fit the best *γ* that minimizes JSD_Radius_, JSD of *p*_r_. For the grid search, we explore *p* with dividing 0 to 1 into 51 bins (bin width = 0.02), *k* with dividing 0–3 into 121 bins (bin width = 0.025), and *γ* with dividing 0–5 into 201 bins (bin width = 0.025). We repeat the simulation 10 times and average the estimated model parameters.

Our model successfully reconstructs the flow matrix *T*_model_, all distributions, and the retention of attachment to a location (Fig. 5b–d). Figure 5b shows the flow matrix from the model, *T*^model^. A matrix distance between *T*^model^ and *T*^data^ measured by the Frobenius norm *d*_T_ = ∣∣*T*^model^−*T*^data^∣∣_F_ is 0.03, indicating the model reproduces the flows across the recreational hierarchy. Although there are gaps in the first bin of the generated distributions and the home ratio, our model captures the overall patterns of individual movements well. It is difficult to model outliers who rarely move to other places. Furthermore, we compare the simulated reservation counts from the model and the actual reservation counts and find that the model also explains the reservation count well for both the pre- and post-COVID-19 periods (Supplementary Fig. S7).

To better understand the effects of these factors on recreational gatherings, we examine the variant models that exclude each factor (Supplementary Fig. S8). From the estimated *p*, *k*, and *γ* for the best model, we construct the variant models by changing the target parameter while keeping other parameters the same. For the model without recreational hierarchy, we simulate the model with *k* = 0. In this model, an agent does not consider recreational hierarchy, and this change results in a collapse of the model in the flow matrix (*d*_T_ = 0.35; Supplementary Fig. S8a) and *p*_h_ (Supplementary Fig. S8b). Similarly, we try the model without geography with *γ* = 0 where an agent does not take into account geographical distance. This model still produces a comparable result on the hierarchy (*d*_T_ = 0.06) and *p*_h_, while *p*_r_ is totally collapsed as expected (Supplementary Fig. S8e). Lastly, we build the model without attachment to a location with *i* = 1. In this model, the likelihood that an agent explores a place is always *p* so that the likelihood does not depend on the iteration. The model without the attachment reproduces the hierarchy (*d*_T_ = 0.04), weakly collapses in *p*_h_ and *p*_r_, but does not reproduce the retention of attachment to a location (Supplementary Fig. S8i). In short, recreational hierarchy, geographical distance, and attachment to a location are indispensable factors of urban recreational gatherings in Seoul.

### An external shock influences recreational gatherings

**Fig. 6:**
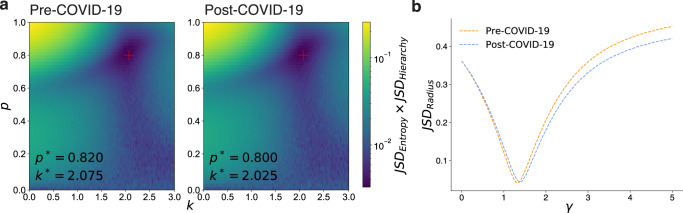
Model parameter estimation. **a** JSD_Entrop*y*_ × JSD_Hierarchy_ varied by the model parameters *p* and *k*. Bottom annotated p^*^ and k^*^ are the best parameters for each period. **b** JSD_Radius_ varied by the model parameter *γ*. We change *γ* while keeping the best *p* and *k* from (**a**).

We explore the influence of the COVID-19 outbreak on individual movements for recreational activities by comparing the best model result for each period. We show the JSD_Entropy_ and JSD_Hierarchy_ varied by model parameters *p* and *k* in Fig. 6a and the JSD_Radius_ varied by model parameter *γ* in Fig. 6b. For both periods, the overall fitness landscape does not change, while the optimal point does. In response to the COVID-19 pandemic, the likelihood of finding places *p* decreases (*p*_pre_ = 0.820 > *p*_post_ = 0.800), indicating people prefer to stay in familiar places. In addition, the tendency to explore a high-level place decreases (*k*_pre_ = 2.075 > *k*_post_ = 2.025), and people become reluctant to travel far from their recreational homes (*γ*_pre_ = 1.325 < *γ*_post_ = 1.375). To check how much difference the parameter changes make, we simulated the model with the optimal parameters from the pre-COVID-19 period (*p* = 0.820 and *k* = 2.075) for the post-COVID-19 period with the assumption that user behaviors do not change. We checked that JSD_Entropy_ × JSD_Hierarch*y*_ of this model increases by 7% compared to our optimal model. Similarly, if we simulate the model with the previous optimal *γ* from the pre-COVID-19 period, JSD_Radius_ increases by 16%.

The differences in the estimated parameters between the two periods are not subtle, and the model’s goodness of fit is sensitive to the parameter changes. In Fig. 6a, if *p* increases or decreases by 0.02 from the estimated optimal point p^*^, JSD_Entropy_ × JSD_Hierarchy_ increases by a factor of 1.058 and 1.080, respectively. If *k* increases or decreases by 0.025 from the estimated optimal point k^*^, JSD_Entropy_ × JSD_Hierarchy_ increases by a factor of 1.119 and 1.057, respectively. Similarly, if *γ* increases or decreases by 0.025 from the estimated optimal point γ^*^, JSD_Radius_ increases by a factor of 1.022, 1.024, respectively. Also, we would like to note that the goodness of fit’s standard deviation for ten repetitions is an order of magnitude smaller than the average value of goodness of fit, implying our simulation results are robust to random errors.

Intuitively, before the pandemic, people prefer to visit popular places as they have fewer restrictions on geographic distance. However, during the pandemic, people chose familiar (high *p*), relatively less popular (low *k*), and closer places (large *γ*) from their recreational homes. Low *k* would reflect the behaviors of avoiding dense areas to prevent exposure to COVID-19, and large *γ* would be associated with the tendency not to take public transportation. After the outbreak, the public transportation system usage in Seoul declined sharply: −26.5% for the bus, −27.5% for the subway in 2020 compared to the previous year, according to a report from the Korean Ministry of Land, Infrastructure, and Transport (KMOLIT) (2021). Furthermore, these changes imply the effect of COVID-19 on urban inequality. As people avert crowded places but choose less popular places, the concentration of activities at the highest level is dissolved. However, the number of visitations at low hierarchy levels (*l* ≤ 6) also decreases (Supplementary Fig. S9) because the probability of exploring places *p*_*i*_ quickly converges to zero by iterations.

## Discussion

In this paper, we quantify the characteristics of recreational visitations with three factors: recreational hierarchy, geographical distance, and attachment to a location. Leveraging our findings, we develop a model that successfully reconstructs and explains empirical patterns found in Seoul, Republic of Korea. We show that the COVID-19 pandemic led people to be less likely to visit different levels in the hierarchy. They prefer familiar, less popular, and closer locations. Furthermore, we suggest a possible mechanism to explain the worsening inequality with the model parameters *p* and *k* simultaneously.

Our study has several limitations. First, agents start from the empty reservation history and find a place by the model mechanisms. In reality, each individual could have past reservation records and find a place depending on the given history. Second, we use the geographic distance between cells, while the urban transportation systems can distort the distance. Third, our data are strongly correlated with mobility inflows. Therefore, changes in motel booking behaviors would explain changes in recreational activities. Considering diverse layers and their interactions can deeply enrich the understanding of the urban activity. Fourth, our findings on behavioral changes could be the combination of voluntary willingness to avoid physical contact and public health guidelines such as social distancing policies, operating hours restrictions, and maximum occupancy restrictions. People might gather in less popular places to avoid waiting because many facilities could handle fewer people than before due to capacity limits. Additionally, they might prefer to gather in closer locations to return home without spending much time on public transportation. It would be interesting to decompose the effects of these factors with high-resolution data and advanced models. Fifth, our findings on the behavioral changes would be mainly led by low- and middle-income populations, the primary users of the platform we studied.

Despite these limitations, our study enhances the understanding of urban human activities and would help design effective public health policies considering individual movements around home areas. Practically, our model can be a simulation tool to prepare for unexpected future events that may affect human behaviors. With our model, academic and industry researchers can also tackle inequality issues stemming from behavioral changes across the urban hierarchy.

## Supplementary information

Supplementary Information

## Data Availability

Due to privacy concerns, the accommodation reservation data we used cannot be shared publicly. The Seoul mobility data is publicly available. The scripts used in this analysis can be found at https://github.com/jisungyoon/hotspot.
