# Supplementary material for: COVID-19 confines recreational gatherings in Seoul to familiar, less crowded, and neighboring urban areas
Source: Humanit Soc Sci Commun. 2022 Sep 23;9(1):330. doi: 10.1057/s41599-022-01349-4 (PMC9510209; doi:10.1057/s41599-022-01349-4)
Supplement: Supplementary file 1 — Supplementary Information [file 41599_2022_1349_MOESM1_ESM.pdf]

Supplementary Information: COVID-19 confines recreational gatherings in Seoul to familiar, less crowded, and neighboring urban areas

September 2, 2022

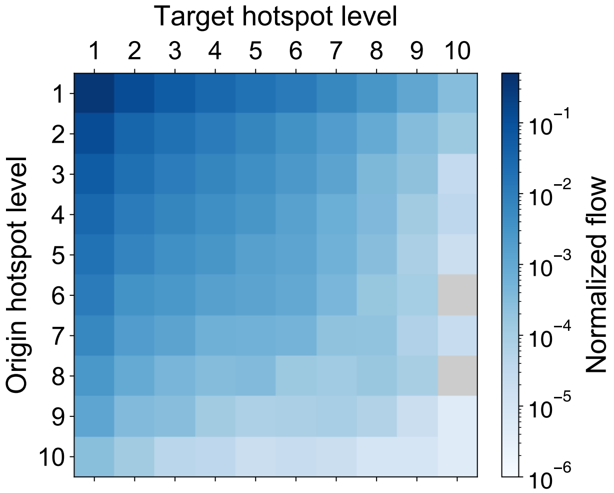

Figure S1: The flow matrix T^{data} for the post-COVID-19 period. As same as Fig. 1 in the main report, we exclude the trips within the same cell. There are no transition records for the gray cells.

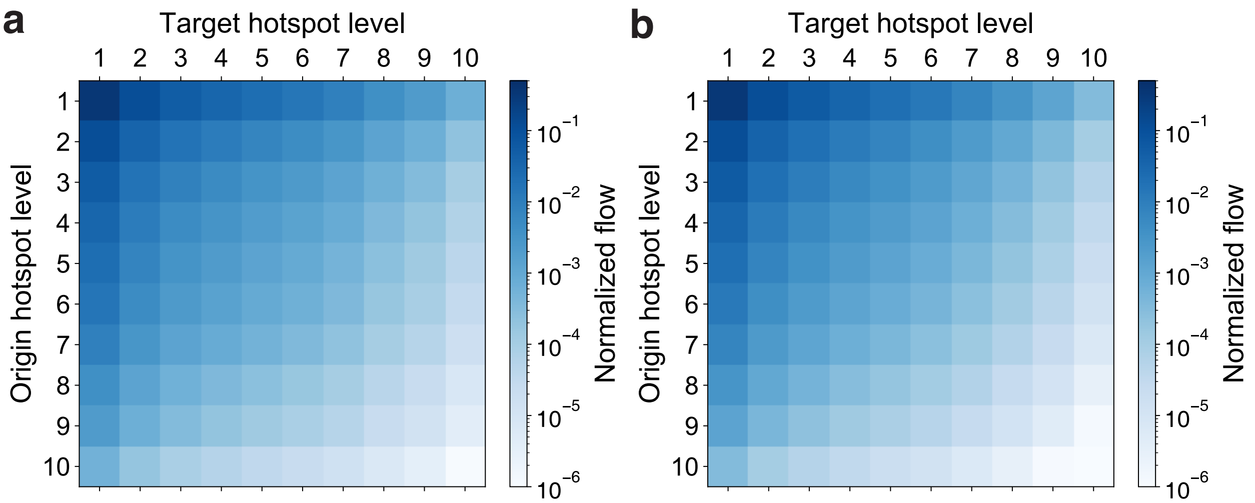

Figure S2: The flow matrix of the null model, T^{null}, for the (a) pre-COVID-19 period and the (b) post-COVID-19 period.

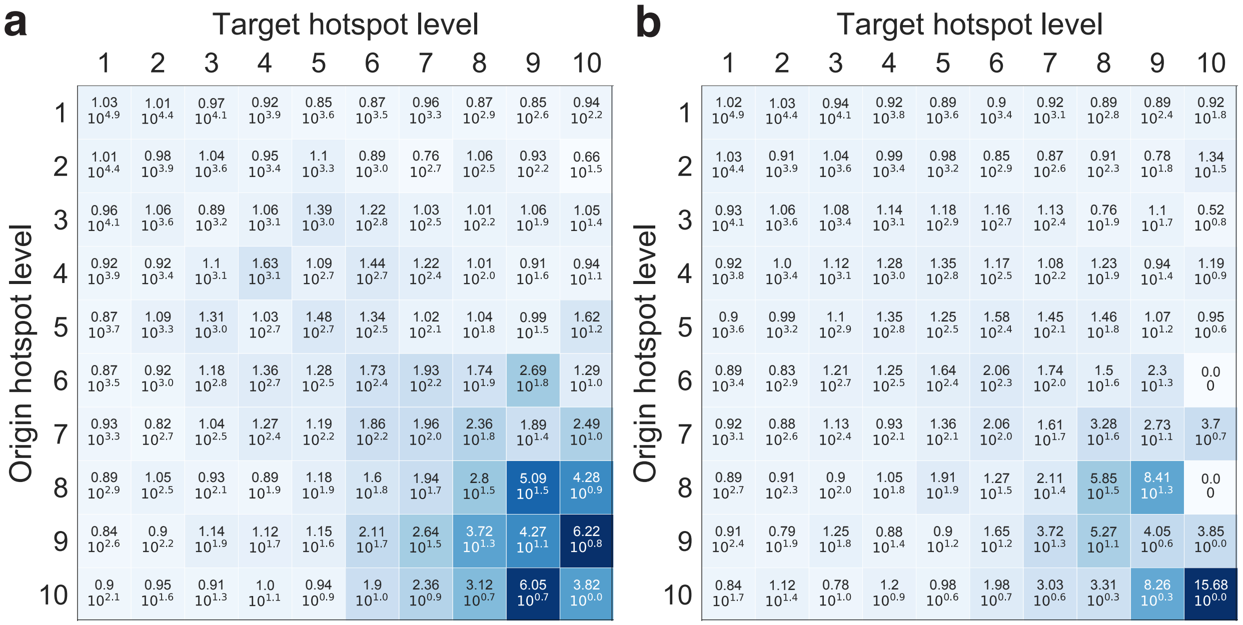

Figure S3: The ratio matrix T^{data}/T^{null} in the (a) pre-COVID-19 period and the (b) post-COVID-19 period. In each cell, upper annotated number is T_{data}/T_{null} and below annotated number is the total number of transitions in the data.

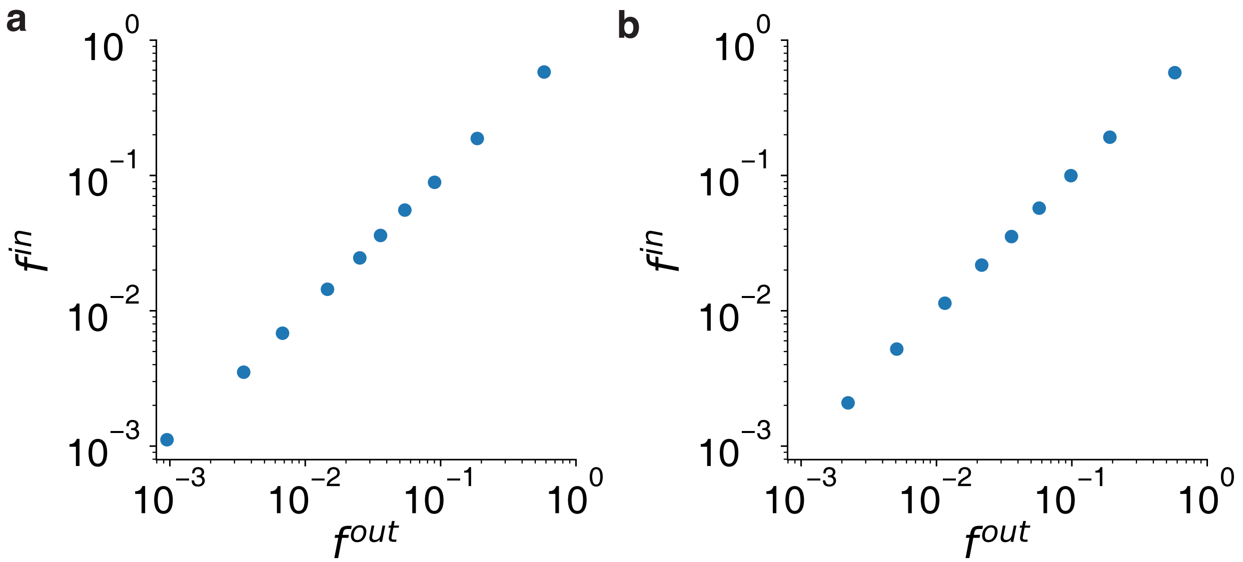

Figure S4: Transition outflows and inflows in (a) the pre-COVID-19 period and (b) the post-COVID-19 period. The infow is defined as $f_i^{in} = \sum_{k=1}^{L} T_{ki}$ and outflow is defined as $f_i^{out} = \sum_{k=1}^{L} T_{ik}$ where L is the total number of hotspot level. f^{in} and f^{out} is almost symmetry for both periods ($R^2 > 0.999$).

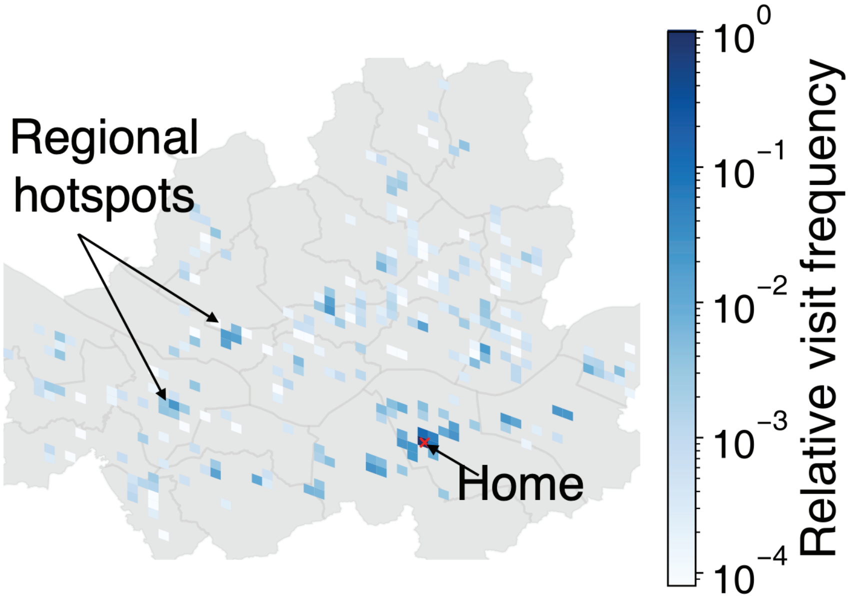

Figure S5: Urban hierarchy leads people to move farther than expected. We collect the trajectories of which recreational home cells are near the Gangnam area (red cross, hotspot level 1), which is one of popular regions in Seoul. Relative visit frequency decays with the geographic distance from the home cell, but the geographic distance cannot explain the pattern near regional hotspots.

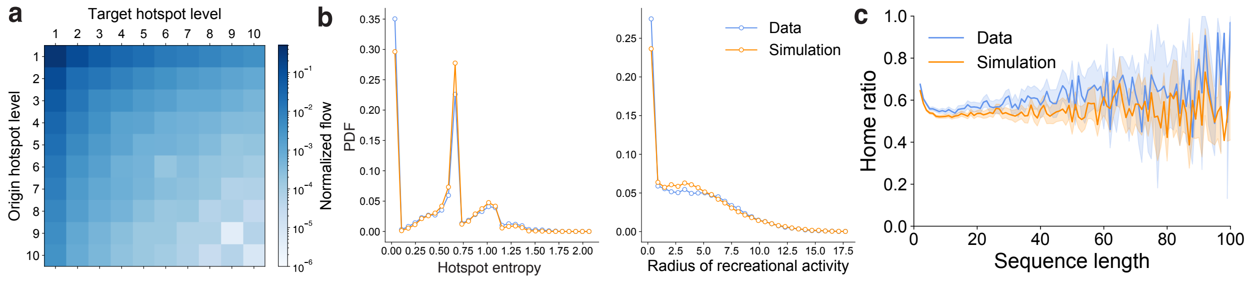

Figure S6: The best model result for the post-COIVD-19 period (a) The flow matrix T^{model} of the best model, $d_T = ||T^{model} - T^{data}||_F$ is 0.02. **(b) The hotspot entropy distribution p_h (left) and the distribution of the radius of recreational activities p_r (right).** Blue lines are the empirical distributions, and orange lines are the simulation results. **(c) Home ratios by the length of trajectory.**

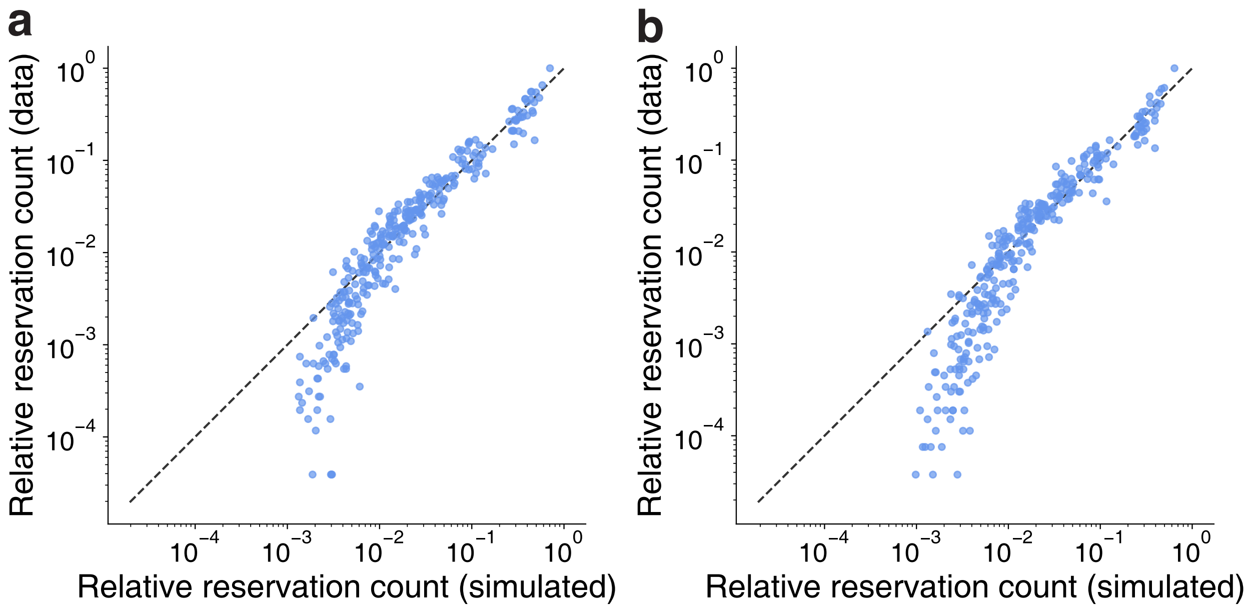

Figure S7: Cell-level comparisons between actual and simulated reservation counts for the (a) pre-COVID-19 period and the (b) post-COVID-19 period. Overall, our model simulates the cell-level reservation count well for both periods. For the data privacy concern, we normalize the reservation count with the maximum reservation count of the actual data set.

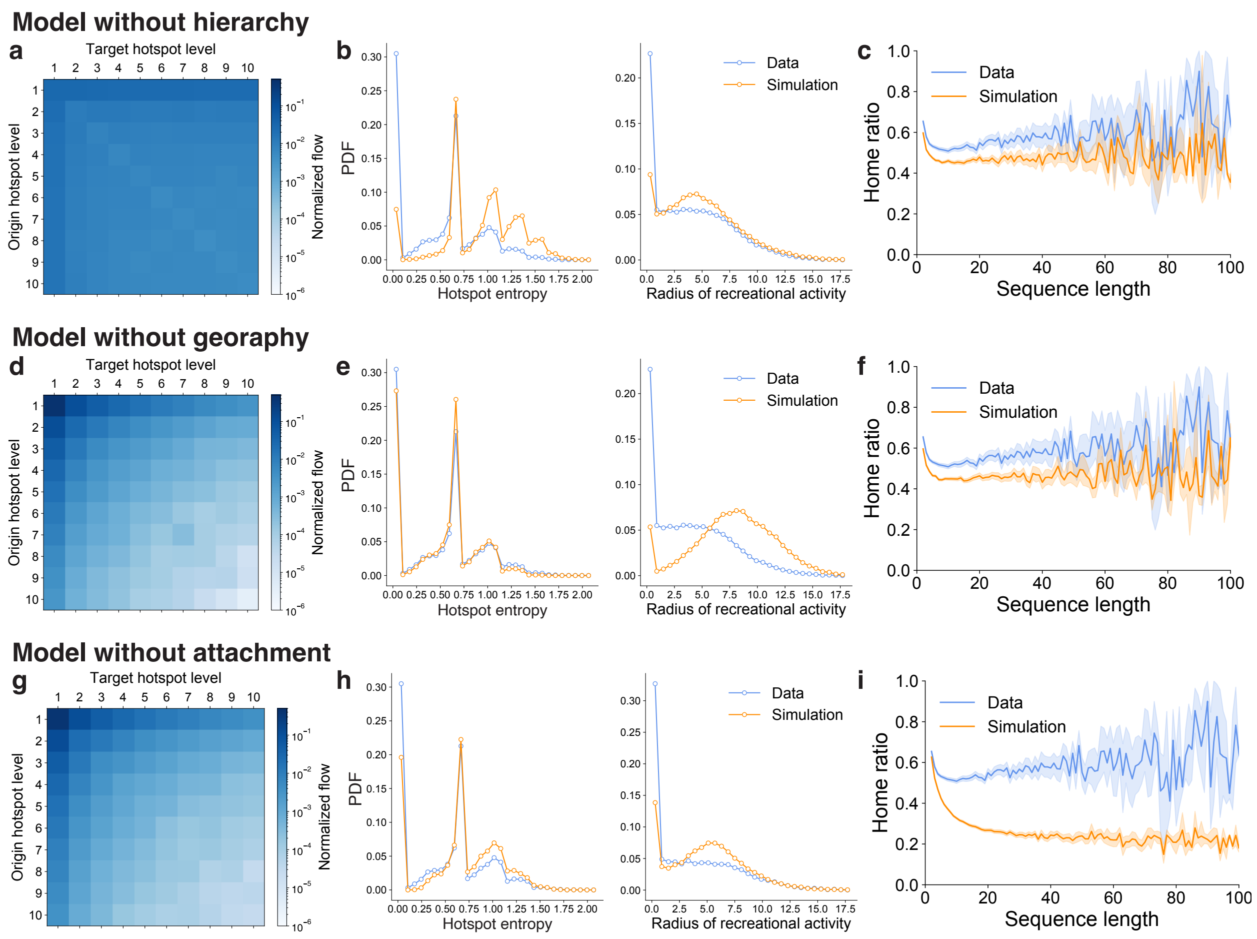

Figure S8: Variant model results for the pre-COIVD-19 period. (a)-(c) The model without urban hierarchy. (d)-(f) The model without geography. (g)-(h) The model without attachment to a location. The figures in the first column are the flow matrices from each model T^{model}. The figures in the second column are the hotspot entropy distributions p_h^{model} (left) and the radius of recreational activities distributions p_r^{model} (right) from each model. Blue lines are the empirical distributions, and orange lines are the simulation results. Lastly, the figures in the third column are home ratios by sequence length. The overall pattern is similar to the pattern in the post-COVID-19 period.

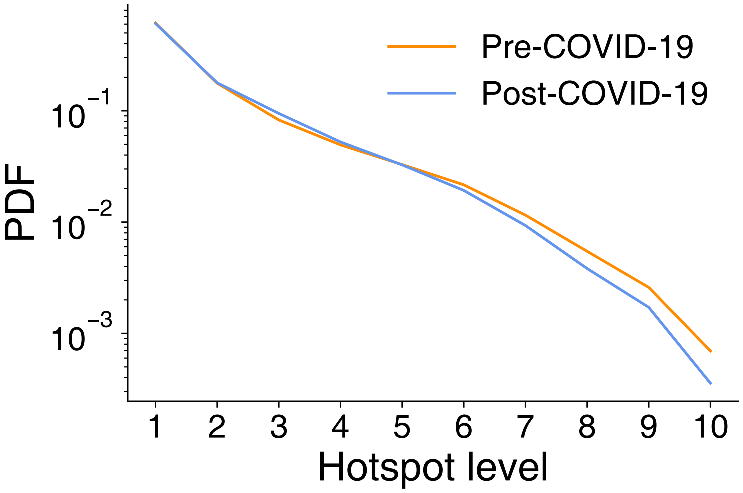

Figure S9: The simulated reservation count distribution by the hotspot level p_ℓ. The model successfully explains the decentralization of human urban activities and the worsening inequality of the urban areas in Seoul.
